# Metazoan Parasite Vaccines: Present Status and Future Prospects

**DOI:** 10.3389/fcimb.2018.00067

**Published:** 2018-03-13

**Authors:** Christian Stutzer, Sabine A. Richards, Mariette Ferreira, Samantha Baron, Christine Maritz-Olivier

**Affiliations:** Tick Vaccine Group, Department of Genetics, University of Pretoria, Pretoria, South Africa

**Keywords:** parasites, vaccines, parasite control, antigen identification, systems biology, OMICS techniques, vaccine development

## Abstract

Eukaryotic parasites and pathogens continue to cause some of the most detrimental and difficult to treat diseases (or disease states) in both humans and animals, while also continuously expanding into non-endemic countries. Combined with the ever growing number of reports on drug-resistance and the lack of effective treatment programs for many metazoan diseases, the impact that these organisms will have on quality of life remain a global challenge. Vaccination as an effective prophylactic treatment has been demonstrated for well over 200 years for bacterial and viral diseases. From the earliest variolation procedures to the cutting edge technologies employed today, many protective preparations have been successfully developed for use in both medical and veterinary applications. In spite of the successes of these applications in the discovery of subunit vaccines against prokaryotic pathogens, not many targets have been successfully developed into vaccines directed against metazoan parasites. With the current increase in -omics technologies and metadata for eukaryotic parasites, target discovery for vaccine development can be expedited. However, a good understanding of the host/vector/pathogen interface is needed to understand the underlying biological, biochemical and immunological components that will confer a protective response in the host animal. Therefore, systems biology is rapidly coming of age in the pursuit of effective parasite vaccines. Despite the difficulties, a number of approaches have been developed and applied to parasitic helminths and arthropods. This review will focus on key aspects of vaccine development that require attention in the battle against these metazoan parasites, as well as successes in the field of vaccine development for helminthiases and ectoparasites. Lastly, we propose future direction of applying successes in pursuit of next generation vaccines.

## Introduction

It is projected that the combined populations of Asia and Africa will constitute 9 billion of the 11 billion global population by 2100 (FAO, 2017). With ever increasing urbanization, there is an apparent concomitant shift from low agricultural labor productivity to higher labor productivity in services and manufacturing for many developing countries of sub-Saharan Africa, Asia and Latin America (Nations, 2015; Diao et al., 2017). At the same time, the global demand for animal and animal-derived products will increase with sustainable and efficient production practices becoming paramount to ensure food security and food safety (Godfray et al., 2010; Herrero and Thornton, 2013; Allen and Prosperi, 2016; Little et al., 2016; OECD/FAO, 2016). This is, however, a challenge when sustainable growth in agricultural productivity is hindered by the inevitable degradation of natural resources (i.e., land and water), losses in biodiversity, as well as the spread of trans-boundary plant and animal pests and diseases (Garnett et al., 2013; Fry et al., 2016; Pekel et al., 2016; FAO, 2017; Watts et al., 2017). Introduction and/or resurgence of pests and diseases within endemic and non-endemic regions of the world will not only threaten public health and welfare directly, but also indirectly through veterinary health that affects animal-derived commodities needed for nutrition (improving health and growth) and generation of wealth (to relieve poverty) (De Magalhaes and Santaeulalia-Llopis, 2015; Cable et al., 2017). Moreover, as human and animal contact increases through urbanization the potential for transmission/contraction of debilitating or lethal zoonotic diseases is a serious global concern (Polley, 2005; Mackenstedt et al., 2015; Cable et al., 2017).

Pathogens (including some parasites) continue to cause some of the most detrimental and difficult to treat diseases (or disease states) in both humans and animals. Around 17 neglected tropical diseases (NTD) that affect more than a billion people globally have been identified by the World Health Organization (WHO) as priority strategic areas for development of effective control, elimination or eradication programs (WHO, 2012). Of these, 11 are caused by internal eukaryotic parasites or pathogens (http://www.who.int/neglected_diseases/en/)^1^. (Hotez et al., 2016). In addition, around 116 animal (terrestrial and aquatic) diseases, infections or infestations are currently listed as notifiable by the World Organization for Animal Health (OIE) (http://www.oie.int/en/animal-health-in-the-world/oie-listed-diseases-2017), of which some 23 involve eukaryotic parasites or pathogens of agro-veterinary importance (Stentiford et al., 2014). Many of the most important medical and veterinary diseases are vector-borne and/or transferred through animal reservoir hosts (Gubler, 2009; Torgerson, 2013; Wilson et al., 2017), which is exaggerated by poor socioeconomic stability and anthropogenic factors leading to the persistence and/or expansion of parasites into non-endemic areas and associated increases in the agro-developmental burden of developing and poverty stricken regions of the world (Torgerson, 2013; Nii-Trebi, 2017).

In the case of metazoan parasites, prophylactic prevention and treatment relies heavily on antiparasitic drugs and/or chemical control strategies (such as topical dips and sprays) to relieve parasite burden and vector-borne infection rates (Torgerson, 2013; Andrews et al., 2014). Unfortunately, abuse and misuse of such antiparasitics can often have unwanted side effects and prolonged persistence in treated hosts leading to contamination of animal-derived products and environmental resources (Boxall, 2004; Aktar et al., 2009). Though resistance to chemotherapeutics varies widely depending on the biological and ecological complexity of each metazoan parasite, there is enough indication of step-wise gains in resistance for several endo (e.g., helminths) and ectoparasites (e.g., arthropods such as ticks and other biting insects) of global importance (Benz et al., 1989; Jones et al., 1992; Rust and Dryden, 1997; Coles, 1998; Sibley and Hunt, 2003; Vercruysse et al., 2007; Abbas et al., 2014; Liu et al., 2014; Benelli, 2015; Geurden et al., 2015; McNair, 2015; Mougabure-Cueto and Picollo, 2015; Genta et al., 2016; Van Wieren et al., 2016). Moreover, whether existing compounds are used correctly, new drugs are developed and/or restricted use is implemented, eventual selection of resistant populations are inevitable (Molento, 2009). Therefore, complimentary strategies are needed to limit the selection pressures leading to resistance. To date, vaccines have been included successfully as part of integrated pest and disease management programs for both humans and animals representing a promising approach for the future control of endo- and ectoparasites (Lombard et al., 2007; Vercruysse et al., 2007; Mariner et al., 2012; Joice and Lipsitch, 2013; Bottazzi, 2015; Hussein et al., 2015).

This review aims to summarize and highlight some of the successful approaches in developing metazoan parasitic vaccines directed at both endo- and ectoparasites, and to garner lessons from these endeavors that can guide us in the development of the next generation of vaccines.

## Endoparasite vaccines: helminthiases

The largest complement of disease causing parasites of medical and veterinary importance are endo-parasitic, with helminthiases being a major contributor (Hotez et al., 2016), causing a number of chronic and/or debilitating diseases that can impede normal physical and cognitive development, to which children especially are susceptible (Briggs et al., 2016; Hotez et al., 2016). Mass anti-helminthic drug administrations (MDA), such as praziquantel for schistosomiasis and albendazole or mebendazole for soil-transmitted helminthiases (STH), are the main public health interventions prescribed to control morbidity in endemic countries (WHO, 2012), but treatment is marred by the specter of acquired chemical resistance (Sutherland and Leathwick, 2011; Keenan et al., 2013; Geurden et al., 2015). Vaccination as part of an integrated veterinary and public health strategy has been proposed for helminth control and several vaccine candidates are currently in development and/or available for treating helminthic infections (Supplementary Table 1) (Matthews et al., 2016; Molehin et al., 2016; Tebeje et al., 2016). In the following sections selected current lead antigens used in medical and veterinary vaccines against helminthiases will be discussed.

### Trematodes and soil-transmitted helminths (STH)

#### Schistosomiasis

Schistosoma trematodes are freshwater snail-transmitted dimorphic parasitic flatworms that circulate in the bloodstream of mammalian hosts causing a debilitating intravascular disease known as schistosomiasis (or bilharzia) with *Schistosoma haematobium* and *S. mansoni* as the main etiological agents (Adenowo et al., 2015; Haçariz and Sayers, 2016; Molehin et al., 2016; Nii-Trebi, 2017). Currently, some 219 million people are exposed to schistosomiasis in 52 countries of which ~90% of the estimated cases in 2003 occurred in Africa (Wikel, 1999; Steinmann et al., 2006; Adenowo et al., 2015). To date, a number of studies have been conducted and candidate proteins for novel vaccines and diagnostic tools identified (Fonseca et al., 2012; Ludolf et al., 2014; Van Der Ree and Mutapi, 2015; Haçariz and Sayers, 2016; Molehin et al., 2016; Hinz et al., 2017; Homann et al., 2017).

Proof-of-concept for vaccination against Schistosoma trematodes was established in mice and non-human primates immunized with native irradiated cercariae, conferring some 80% protection against schistosomula challenge (Hagan et al., 1995; Coulson, 1997; Coulson and Wilson, 1997). Attenuated preparations were deemed unfeasible as a human cercarial vaccine and subunit vaccine development was pursued (Molehin et al., 2016). Targets for current lead subunit vaccines were primarily identified using classical biochemical techniques from parasite protein fractions (e.g., *S. mansoni* 28 kDa glutathione S-transferase and 14 kDa fatty-acid-binding protein antigens) (Hagan et al., 1995). These targets are mostly excretory/secretory (ES) surface molecules from tegumental membranes of the migrating schistosomulum stages (affecting parasite invasion), as well as the adult females (affecting parasite survival and fecundity) (Hagan et al., 1995; Ricciardi and Ndao, 2015). Current advances in high-throughput technologies have expedited antigen identification for important schistosome species, but translation from basic research to clinical and field evaluations is still lacking. Only a few recombinant targets are currently in varying stages of clinical and commercial development (Merrifield et al., 2016; Molehin et al., 2016; Tebeje et al., 2016).

A common *S. haematobium* 28 kDa glutathione S-transferase (Sh28GST) protein, with a wide distribution in parenchymal tissues and tegumental structures of immature and adult worms (Balloul et al., 1985, 1987a; Porchet et al., 1994), is currently being evaluated in phase III clinical trials as Bilhvax for treating urinary schistisomiasis (Supplementary Table 1) (Riveau et al., 2012; Ricciardi and Ndao, 2015; Molehin et al., 2016). This antigen was originally cloned, crystallized and tested with promising results against *S. mansoni* (Balloul et al., 1987b; Trottein et al., 1992; Hagan et al., 1995). The precise biological role of this antigen is however not fully elucidated, but evidence for its involvement in parasite cellular detoxification and host immune regulation has been presented (Huang et al., 2012). Since GST proteins also play essential roles in parasite resistance to chemotherapy (Huang et al., 2012; Joice and Lipsitch, 2013), a combinatorial approach using a GST-directed vaccine and chemotherapies targeting parasite detoxification processes (e.g., Praziquantel or PZQ) could have excellent therapeutic potential (Huang et al., 2012). Unfortunately no further data is publically available for phase III clinical evaluations since 2009 and protection efficacy studies in human subjects remain elusive. This antigen has however been patented and applications for evaluation in separate phase II clinical trials against Crohn's inflammatory autoimmune disease is in progress (Supplementary Table 1).

For intestinal schistosomiasis, a recombinant *S. mansoni* 14 kDa monovalent fatty acid-binding protein (Sm14) (Moser et al., 1991; Tendler and Simpson, 2008; Coler et al., 2011), is currently in the final stages of phase II field trial testing in adult male subjects from endemic regions in Africa and Brazil (Supplementary Table 1) (Ricciardi and Ndao, 2015; Tendler et al., 2015; Santini-Oliveira et al., 2016). This antigen also shows promise as a dual-purpose helminthic vaccine against veterinary fasciolosis by conferring complete protection in mice challenged with *Fasciola hepatica* (67% for *S. mansoni*) (Tendler et al., 1996) and vice versa with a similar native protein isolated from *Fasciola hepatica* (nFh12) that cross-protected mice against *S. mansoni* infection (>80% efficacy) (Vicente et al., 2016). This study is currently active and promising results could eventually lead to the first human antihelmintic vaccine.

An additional *S. mansoni* 9 kDa tetraspanin surface protein (Sm-TSP-2) is also showing promise in phase I human clinical trials (Supplementary Table 1) (Tran et al., 2006; Pearson et al., 2012). Previous pre-clinical immunization studies in mice indicated a reduction in adult parasite and liver egg burdens of 57 and 64%, respectively, following challenge with *S. mansoni* (Tran et al., 2006). A similar tetraspanin loop protein (TSP-LEL) has also been successfully evaluated in mice and non-human primates (~95% and >88% protection, respectively) as a multivalent fusion protein (rBmHAT vaccine) as a treatment against human lymphatic filariasis caused by the nematode *Brugia malayi* (Dakshinamoorthy et al., 2013, 2014; Chauhan et al., 2017a). A membrane surface calpain (Sm-p80) involved in parasite membrane renewal, is also showing promise as a lead candidate (Siddiqui et al., 1993; Ahmad et al., 2009) with proof-of-concept studies completed in animal models (including non-human primates) showing significant cross-species protection against *S. haematobium* and *S. japonicum* (Zhang et al., 2001; Karmakar et al., 2014a,b; Molehin et al., 2016). A recombinant Sm-p80/GLA-SE vaccine is currently trademarked as SchistoShield®, and is going through process development, formulation, stability and potency testing for final international patenting and phase I/II human clinical trials (Supplementary Table 1) (Molehin et al., 2016).

For the zoonotic parasite, *S. japonicum*, mostly transmission blocking vaccines are being pursued to limit parasite transmission and persistence in reservoir animal hosts consequently lowering infection rates in humans (Molehin et al., 2016; Tebeje et al., 2016). A number of candidate antigens (Sj97, Sj23 and SjTPI) shown promise for treatment of *S. japonicum* in domesticated animals (Ohta et al., 2004; Zhu et al., 2004, 2006; Dai et al., 2014, 2015; Jiz et al., 2015; Molehin et al., 2016). However, extensive field evaluations are needed for a commercial vaccine to be developed.

#### Soil-transmitted helminths (STH): hookworm disease

Soil-transmitted helminths (STH) are a group of medically important endoparasites that include: roundworms (e.g., *Ascaris lumbricoides*); whipworms (e.g., *Trichuris trichiura*) and hookworms (*Necator americanus* and *Ancylostoma duodenale*) (WHO, 2016). Currently, approximately two-thirds of the global human disease burden from soil-transmitted nematode infections can be ascribed to hookworm infections and as such, much effort has been expended in the pursuit of a vaccine (Murray et al., 2013). Human hookworm disease, with *N. americanus* and *A. duodenale* as principal disease causing parasites, is an affliction causing severe anemia affecting some 440 million people globally in Asia, sub-Saharan Africa, the Caribbean and Latin America (Hotez et al., 2013; Pullan et al., 2014). Hookworm vaccine development was initially evaluated in the veterinary field by Miller et al. using a native preparation derived from whole irradiated *A. caninum* L3 larvae to immunize 3–4 month old dogs (Miller, 1964). This seminal work indicated a 37% overall vaccine protection with a 91% decrease in fecal egg output that was further optimized to confer more than 80% protection depending on the route of administration (Miller, 1964, 1965) resulting in a commercialized vaccine in the United States in 1973 for canines. But this vaccine was discontinued 2 years later due to various limitations recorded at the time that included cost, storage and stability, as well as the lack of sterilizing immunity (Miller, 1978; Schneider et al., 2011).

For further subunit vaccine development, the infective larval L3 stage was explored, since many stages-specific targets are produced that are essential for host invasion, modulation of host immunity and parasite establishment. The latter includes all of the released excretory-secretory (or ES) products (Hawdon et al., 1996; Bethony et al., 2008; Bottazzi, 2015), of which a tissue invasion-related metalloprotease (Zhan et al., 2002; Williamson et al., 2006) and two Ancylostoma secreted proteins (ASP-1 and ASP-2) were identified from the canine hookworm *A. caninum* (Hawdon et al., 1996, 1999). ASPs are cysteine-rich proteins of unknown function that could be linked to pathogenesis via screening of hyper immune serum from humans living in endemic countries (Brazil and China) detected ASP-2 (Bethony et al., 2005). The ASP-2 antigen was considered a lead candidate for human hookworm vaccine development and promising results were obtained in both animal models and preliminary human clinical vaccination trials (Bethony et al., 2005, 2008; Mendez et al., 2005). However, further human clinical trials using recombinant *N. americanus* ASP-2 (*Na*-ASP-2) antigen were halted in 2008 due to adverse reactions (generalized urticaria) observed in a Brazillian cohort of chronically infected subjects (Schneider et al., 2011; Diemert et al., 2012; Bottazzi, 2015).

Severe immediate-type allergic reactions observed in previously exposed individuals caused investigators to focus more on excreted/secreted adult gut antigens (Hotez et al., 2013). Two *N. americanus* antigens, the aspartic protease haemoglobinase APR-1 and GST-1, shown promise in animal studies (Plieskatt et al., 2012; Hotez et al., 2013; Curti et al., 2014; Bottazzi, 2015).

### Other veterinary helminthiases

#### Gastrointestinal nematodes (GINs)

Both *Ostertagia ostertagi* and *Cooperia oncophora* are the most prevalent gastrointestinal nematodes (GINs) of grazing cattle that often occur as co-infections together and/or with other GINs (Matthews et al., 2016). For *O. ostertagi*, several native vaccine preparations based on excretory/secretory or membrane targets have been shown to reduce worm fecundity by ±50% (Geldhof et al., 2002, 2004; Vercauteren et al., 2004; Claerebout et al., 2005; Meyvis et al., 2007; Geldhof and Knox, 2008; Halliday and Smith, 2010). Activation-associated secreted proteins (ASPs) of unknown function are currently evaluated against both *O. ostertagi* and *C. oncophora* (Geldhof et al., 2003, 2004; Borloo et al., 2013; Vlaminck et al., 2015). Homologous challenge with a double-domain ASP (dd-ASP) protein from *C. oncophora* was shown to reduced cumulative fecal egg counts by 91%, while field trials achieved a 58.8% reduction overall (Vlaminck et al., 2015). Despite promising results for dd-ASP, ASP1 and the polyprotein allergen OPA, a commercial vaccine targeting GINs is hindered by limitations in antigen production and formulation to deliver reproducible results (e.g., ASP1) (Vercauteren et al., 2004; Matthews et al., 2016).

In small ruminants such as sheep, the nematode *Teladorsagia circumcincta* is considered a dominant intestinal parasite within the temperate regions of the world, causing parasitic gastroenteritis (PGE) that has a severe impact on productivity by reducing live weight gain (Nisbet et al., 2016). A multivalent recombinant subunit vaccine was developed by Nisbet and co-workers (Nisbet et al., 2013), consisting of: 4 antigens (an ASP-1, cathepsin F, astacin-like metalloproteinase and an uncharacterized 20 kDa protein) identified from larval excreted/secreted protein extracts; a *T. circumcincta* homolog of an *A. caninum* protective antigen Tc-SAA-1 and 3 larval immunosuppressive molecules (macrophage migration inhibitory factor-1, calcium-dependent apyrase-1 and a TGF beta homolog Tci-TGH-2) (Redmond et al., 2006; Nisbet et al., 2009, 2010a,b, 2011; Smith et al., 2009). Trials performed on lambs showed a reduction in parasite egg production (70 and 58%), reductions in peak egg shedding (92 and 73%) and total worm burdens (75 and 56%) among vaccinated cohorts in two separate trials (Nisbet et al., 2013). Later trials indicated a 45% reduction of egg outputs from vaccinated pregnant ewes during periparturient relaxation in immunity (Nisbet et al., 2016). This vaccine now requires further field evaluations and refinement of components to simplify protein production and enable cost-effective up-scaling for commercial application (Matthews et al., 2016).

The barber's pole worms, *Haemonchus contortus* and *H. placei*, are pathogenic blood-feeding parasites that attach to the abomasum of ruminants (sheep, goats and cattle) causing production losses due to malaise and mortality of susceptible young animals (Matthews et al., 2016). Several gut membrane antigens have been tested in vaccination trials against *H. contortus*, namely: contortin (Hc-PCP1 and Hc-PCP2) (Munn et al., 1987; Geldhof and Knox, 2008); an unknown 100 kDa gut surface antigen (Jasmer et al., 1993, 1996); a microsomal aminopeptidase H11 (Smith et al., 1993), as well as a Haemonchus galactose-containing membrane glycoprotein complex (H-gal-GP) (Smith et al., 1994; Smith and Smith, 1996; Knox et al., 1999). To date, Barbervax® (WormVax, Australia) that contains purified native H-gal-GP and H11 from the guts of adult *H. contortus* is available (Supplementary Table 1) (Bassetto et al., 2014) is available. Unfortunately, subunit vaccines based on H11 and H-gal-GP proteins have been largely unsuccessful in conferring protection, even when producing antigens in *Caenorhabditis elegans* (Cachat et al., 2010; Roberts et al., 2013; Matthews et al., 2016).

Cysteine protease-enriched protein fractions (CPFs) from adult *H. contortus* have been tested in both sheep and goats, with a cathepsin B cysteine proteinase (AC-5) showing promise in immunized lambs (up to 50% reduction in burden and fecundity) (Bakker et al., 2004; Ruiz et al., 2004; De Vries et al., 2009; Molina et al., 2012). Additionally, an adult somatic protein, Hc23 (Domínguez-Toraño et al., 2000; Alunda et al., 2003), is also showing promise achieving an overall reduction of more than 80% in fecal egg counts and parasite burdens in vaccinated lambs (Fawzi et al., 2015). These latter antigens are currently being developed further for a next generation subunit vaccine against *H. contortus*.

Of note is that two other native veterinary vaccines have been commercialized that consist of radiation attenuated L3 larvae for protection against lung worm or lung verminosis infections. These are Bovilis Huskvac® (MSD Animal Health, Ireland) and Difil (Nuclear Research Laboratory of the Indian Veterinary Research Institute, India) designed against *Dictyocaulus viviparous* (bovine lung worm) and *D. filaria*, respectively (Supplementary Table 1) (Sharma et al., 2015).

#### Cestoid parasites

Zoonotic alveolar (AE) and cystic echinococcosis (CE) are recognized neglected diseases caused by *Echinococcus* spp. with a current global burden of some 188,000 (CE) and 18,200 (AE) new human cases per annum (Torgerson et al., 2010, 2015). However, due to the high costs associated with human vaccine development and the relatively low transmission rates of *E. granulosus* in developed countries, commercial incentive is lacking for the production of a human CE vaccine (Lightowlers, 2006; Craig et al., 2017). Therefore, the production of veterinary transmission blocking vaccines are considered a more practical alternative.

Protection was achieved in sheep studies against *E. granulosus* infection following subcutaneous vaccination with either oncospheres (Heath et al., 1981) or fractionated secretory products derived from *in vitro* cultured oncospheres (Osborn and Heath, 1982). Studies performed by Heath and Lawrence (Heath and Lawrence, 1996) identified a protective antigen called EG95, cloned from oncosphere mRNA (Lightowlers et al., 1996), that has conferred up to a 100% protection in experimental immunization trials conducted in seven countries (i.e., Argentina, Australia, Chile, China, Iran, New Zealand, and Romania) (Lightowlers, 2006, 2012; Craig et al., 2017). It was also evaluated for protection against alveolar echincoccosis in sheep caused by *E. multilocularis* with protection ranging between 78.5 and 82.9% (Gauci et al., 2002). Consequently, this antigen is the principal component of the only commercialized vaccine that is registered for use in China (June 2007) and Argentina (February, 2011) (Providean Hidatil EG95®, Tecnovax, Uruguay) (Supplementary Table 1) (Bowman, 2014; Craig et al., 2017).

For alveolar echincoccosis specifically, several additional protective candidates validated in mice have been described including: a recombinant 14-3-3 antigen rE14ζ (97.35% reduction in parasite load following oral egg challenge) (Siles-Lucas et al., 2003, 2008; Craig et al., 2017); the EG95-like protein from *E. multilocularis* EM95 (Gauci et al., 2002), recombinant tetraspanin transmembrane proteins TSP1 and TSP7 (causing a reduction of lesions by 87.5 and 37.6%, respectively) (Dang et al., 2009, 2012), and a metacestode development protein P29 (i.e., rEmP29) that wasfirst identified from *E. granulosus* (up to 75 and 53% reduction in parasite mass and load) (Wang et al., 2009; Boubaker et al., 2015). Therefore, a specific commercial product may stem from these antigens.

Lastly, cysticercosis is a zoonotic tissue infection transmitted between humans (definitive host) and pigs (intermediate hosts) caused by the cestode parasite, *Taenia solium* that is regarded as the most important foodborne parasitic infection and a leading cause of neurological disease in developing countries (Robertson et al., 2013; Lightowlers et al., 2016). A recombinant stage-specific oncosphere surface antigen TSOL18 has been independently tested in both experimental and field trials with a greater than 90% protection conferred in pigs from various localities in Central America, South America and Africa (Lightowlers, 2013; Lightowlers et al., 2016; Lightowlers and Donadeu, 2017). This vaccine is currently the first and only cysticercosis vaccine licensed for commercial production as Cysvax® (Indian Immunologicals Ltd., India) (Supplementary Table 1) (Lightowlers and Donadeu, 2017). Two additional oncosphere-derived proteins, TSOL16 and TSOL45-1A, have shown promise as next generation porcine cysticercosis transmission blocking vaccine candidates with 99.8 and 97.9% protection in vaccinated pigs against *T. solium* cysticerci challenge (Gauci et al., 2012).

Overall, for human helminthiases, no commercial vaccines are currently available with only four recombinant vaccine candidates related to schistosomiasis (*Sh*28GST, Sm14-FABP, Sm-p81, and Sm-TSP-2) being in various processes of clinical development. For the veterinary market, five vaccines have been successfully commercialized that consists of three native (Barbervax®, Huskvac®, and Difil) and two recombinant (Cysvax® and Hidatil EG95®) products. Currently, for veterinary helminth vaccines, it appears that live, inactivated or attenuated vaccines are more successful than recombinant subunit vaccines. This may be due to the need of complex, multi-antigen vaccines that are needed to confer protection to these complex parasites. Live vaccines do however place limitations on the preparation and safety of these vaccines (requiring host animals to produce the parasite material) and an unbroken cold chain that increases costs. In future, subunit vaccines will offer a better solution in terms of safety, stability and production of vaccines. Surprisingly all the antigens mentioned in section Endoparasite Vaccines: Helminthiases, were identified decades before they were commercialized or tested in field or clinical trials, highlighting the arduousness of vaccine development.

## Ectoparasite vaccines: parasitic arthropods

Ectoparasitic arthropods form part of the largest animal phylum of increasing veterinary and medical importance (Mathison and Pritt, 2014; Goddard, 2016). Arthropod-borne pathogens account for more than 20% of all emerging infectious diseases documented between 1940 and 2004 (Jones et al., 2008). However, despite the vast amount of research on vaccine development, there have only been a few commercial successes to date (Supplementary Table 1). Here, we aim to highlight some of the progress and challenges in ectoparasite vaccine development.

### Parasitic flies

Flies of veterinary importance are divided into three major groups, namely the haematophagous biting flies, non-biting nuisance flies and myiasis-causing flies (Pape et al., 2011). Biting flies of veterinary importance include *Haematobia irritans* (horn fly), *H. irritans exigua* (buffalo fly) and *Stomoxys calcitrans* (stable fly) (Pruett, 2002). Horn flies are economically devastating pests of cattle causing severe irritation, reduced milk production, weight loss, substantial blood loss and damage to hides (Byford et al., 1992). Moreover, horn flies are competent vectors of several pathogens including *Stephanofilaria stilesi*, a filarial parasitic nematode of cattle (Hibler, 1966), and several *Staphylococcus* spp. that cause mastitis in dairy heifers (Owens et al., 1998; Gillespie et al., 1999). Stable flies in turn are competent vectors of several pathogenic organisms including viruses [e.g., equine infectious anemia, African horse sickness (AHS) virus and fowl pox], bacteria (e.g., *Brucella* spp. causing brucellosis and *Bacillus anthracis* causing anthrax), protozoa (e.g., *Trypanosoma evansi* the causative agent of Surra), as well as helminths (e.g., nematodes such as *Habronema microstoma* and *Dirofilaria* spp.) (Greenberg, 1973; Turell and Knudson, 1987; Mongoh et al., 2008; Baldacchino et al., 2013).

Research has been conducted to develop new control strategies for biting flies, including vaccines. However, no effective targets have been identified to date (Wijffels et al., 1999; Guerrero et al., 2008; Oyarzún et al., 2008). Only two secreted salivary targets conferring partial protection to horn flies in cattle vaccinations have been identified to date: an anti-thrombin peptide (thrombostasin) (Zhang et al., 2001; Cupp et al., 2004, 2010) and a hematobin (Breijo et al., 2017). With the lack of tools for rapid validation of promising antigens, RNAi has been used despite its inability to translate a phenotype directly into protection. An RNAi study by Torres et al. targeting transcripts of the abdominal tissue of partially fed female horn flies revealed significant mortality and reduced oviposition rates for selected transcript functional groups (Torres et al., 2011). However, off-target effects influenced the results obtained and further optimization is required (Torres et al., 2011; Marr et al., 2014). Currently, no effective candidates are available for biting flies and the rationale for future vaccine development strategies remains to be demonstrated.

### Myiasis-causing flies

Myiasis infections refer to the infestation of a host with the larvae of a range of species with adverse consequences to the host. Myiasis-causing flies are classified into three different groups based on their pathology: cutaneous myiasis-causing skin infections; bot flies that infect the gastrointestinal tract and body orifices of the host; as well as warbles that infect and migrate subcutaneously (Stevens and Wallman, 2006; Stevens et al., 2006).

The majority of research conducted on myiasis-causing flies focused on understanding the pathology and immune reactions caused by sheep blowflies (*Lucilia cuprina* and *L. sericata*) and cattle warble flies (*Hypoderma lineatum* and *H. bovis*). Larvae from *Lucilla* spp. are responsible for cutaneous myiasis, also known as blowfly strike, with a substantial economic impact on the wool industry (Elkington and Mahony, 2007). The immune responses to blowfly strike and vaccine development have been reviewed extensively (Elkington and Mahony, 2007; Sandeman et al., 2014). Many studies have focused on raising protective antibodies against a range of antigens derived from *L. cuprina* larvae including: cuticle proteins (Barrett and Trevella, 1989); whole larvae extracts (East et al., 1992); fractionated larval extracts (Tellam and Eisemann, 1998); excretory and secretory (ES) products (Bowles et al., 1987; Tellam et al., 1994); purified serine-proteases (Tellam et al., 1994) and larval peritrophin membrane proteins (Casu et al., 1997; Tellam and Eisemann, 1998; Colditz et al., 2002). In many instances larval growth was significantly retarded *in vitro* when using antisera raised from immunized animal models. However, no significant protection was conferred against larval infestations during *in vivo* studies. These results were mainly attributed to insufficient levels of IgG produced *in vivo* (2- to 4-fold lower than that used in *in vitro* studies) (Johnston et al., 1992).

As eliciting protective humoral immune responses is challenging, further studies are now focusing on cellular immunity (Sandeman et al., 1986; Bowles et al., 1987, 1996). In this regard, a vaccine formulation containing native antigens derived from larvae, adjuvant (Montanide™ ISA-25) and recombinant ovine interleukin-1β (rovIL-1β) were used for immunization of sheep resulting in significant levels of protection in two consecutive trials (86–67% reduction in strike incidence and 85–31% reduction in larval weight) (Bowles et al., 1996). A fundamental result from this study was that the humoral response did not correlate to the levels of protection, and more significantly that the antibodies derived from the serum are also unlikely to confer protection. Stimulation of type I (IgE-mediated) and type III (Arthus-type) immediate hypersensitivity responses were instead indicated as involved in protection, which was supported by previous observations in blowfly “resistant” sheep (Sandeman et al., 1986; Bowles et al., 1987, 1996). In addition, a 56 kDa excretory/secretory protein from *L. cuprina* larval tissues was shown to inhibit lymphocyte activation, supporting the notion that antibody-mediated immunity is not always sufficient for control of some parasites (Elkington et al., 2009).

In contrast to blowfly infections, it is well known that cattle develop a protective immunity against cattle grub infections, caused by *H. lineatum* (Gingrich, 1980, 1982; Pruett and Kunz, 1996). Increased mortality in larvae was demonstrated during cattle vaccination studies against *H. lineatum* crude larval extracts and culture-derived antigens with some cross-protection induced against *H. bovis* (Baron and Weintruab, 1986). A later study reported a 100% mortality rate for *H. lineatum* following immunization with soluble extracts of third instar fat body using an adjuvant (Colwell, 2011). Additional secreted serine proteinases (hypodermins HyA, HyB, and HyC) were also tested in cattle trials and showed a significant decrease in the amount of developing pupae (Baron and Colwell, 1991). Hypodermin A appears to be the most promising lead antigen and is implicated in the downregulation of host lymphocyte proliferation (Panadero et al., 2009) and cytokine responses *in vivo* (Nicolas-Gaulard et al., 1995). However, to the best of our knowledge no large-scale evaluations have been performed to test the efficacy of Hypodermin A under field conditions.

### Mosquitoes

With almost 1 million human deaths associated with mosquito borne illnesses every year (Caraballo and King, 2014), mosquitoes represent a major threat to human health. Diseases involved include malaria (caused by *Plasmodium* species) and filariasis (cause by filarial nematodes) (Tolle, 2009). Mosquitoes are also well known vectors of viruses including Dengue, West Nile, Chikungunya, Yellow fever and Japanese encephalitis (Tolle, 2009). Current research is focusing on the development of vector-directed vaccines and/or transmission blocking vaccines (targeting the pathogen) to reduce disease occurrence in human and animal hosts. The latter fall outside of the scope of this review. However, vector control is a vital part of disease reduction, since the number of competent vectors can directly affect the incidence of disease.

With regard to blocking of mosquito antigens to control Plasmodium transmission, a lead candidate is the mosquito aminopeptidase N1 (APN1, located on the mosquito midgut luminal surface) that is suggested to be involved in Plasmodium ookinete invasion of the midgut (Dinglasan et al., 2007). Varying transmission blocking activities have been reported with the *Anopheles gambiae* APN1 antigen produced *in situ* using viral vectors, all largely unsuccessful (Kapulu et al., 2015). In contrast, antibodies targeting *An*APN1 in various *Anopheles* species were found to inhibit transmission of several *Plasmodium* species (Dinglasan et al., 2007; Mathias et al., 2012; Armistead et al., 2014). A single protective, highly conserved epitope has consequently been identified for *An*APN1 that has been tested both in murine and non-human primate models with great success (Armistead et al., 2014). A number of additional mosquito antigens have been identified to control Plasmodium transmission, including numerous *A. aegypti* midgut proteases (Shahabuddin et al., 1995; Molina-Cruz et al., 2005; Lavazec et al., 2007), an *An. gambiae* midgut chitinase propeptide (Bhatnagar et al., 2003) a 12 amino acid peptide (SM1- salivary gland and midgut peptide 1) from *An. stephensi* (Ghosh et al., 2001) and a putative transcription factor akrin (or subolesin) (Moreno-Cid et al., 2010, 2013). All of the latter remains to be validated before any definite prediction with regards to their potential as commercial vaccine antigens can be made. One shortfall requiring attention is the need for improved immunogenicity of antigens and longevity of immune responses raised in the respective hosts (Sinden, 2017).

As arthropod saliva is known to mediate host immunity and in doing so aid the transmission of disease causing agents (Titus et al., 2006), it remains a research focus area. A breakthrough with regards to mosquito vaccines has been made in recent years with a multi-antigen vaccine being tested in clinical phase I trials at the moment (Supplementary Table 1). Based on an interview with one of the main researchers, salivary proteins were tested in several groups based on weights with main focus on 20–40 kDa sized proteins with four subsequently chosen based on their occurrence in several types of mosquitoes. The AGS-v vaccine is suggested to not only reduce parasite transmission, but potentially reduce the lifespan of the mosquito itself (Mole, 2017; Unwin, 2017). However, in spite of the significant progress made in recent years, the development of a vaccine targeting mosquito antigens has been slow. Knowledge regarding the mechanism of *Plasmodium* ookinete recognition and invasion of the midgut epithelium, as well as parasite defenses against host and vector immune factors is still lacking considering that successful transmission blocking vaccines are assumed to target accessible vector midgut antigens via antibodies subsequently interfering with their function (Sinden, 2017).

### Lice

Lice are apterous obligate ectoparasites, belonging to the order Phthiraptera, of which only a small fraction is of economic importance. Currently, the majority of vaccine research is dedicated to the aquatic sea lice *Lepeophtheirus salmonis* and *Caligus rogercresseyi* (Pike and Wadsworth, 1999; Costello, 2006). A recombinant peptide-based subunit vaccine against *C. rogercresseyi*, Providean Aquatec Sea Lice® (Tecnovax S.A., Argentina), is currently commercially available (Supplementary Table 1) (Villegas, 2015). However, as aquatic parasites falls outside the scope of this review, they will not be discussed further.

### Acarines

#### Mites

*Sarcoptes scabiei* is a parasitic mite responsible for sarcoptic mange and scabies in both animal and human hosts (Mellanby, 1941, 1944). The invasive nature of this mite enables it to burrow down into the skin of its host resulting in inflammation and intense hypersensitivity responses that can result in severe secondary bacterial infections (Walton et al., 2004). Currently no vaccine against scabies is available and limited advances have been made in the field to date (Arlian and Morgan, 2017). The most promising vaccination studies performed thus far include immunization of rabbits with extracts of the house dust mite which elicited only partial protection (Arlian et al., 1995).

Acquired resistance to *Sarcoptes scabiei var. canis* has been described in canines that was previously infested and then again challenged under laboratory conditions. These animals had increased IgG1, IgG2, IgM antibodies (Arlian et al., 1996) that can now be used in follow-up studies to gain insight into drivers of protective immunity. In contrast, sheep that were infected with *Sarcoptes Scabiei var. ovis* only acquired partial protection despite them also inducing IgG and IgE antibody responses (Rodríguez-Cadenas et al., 2010).

The sheep scab mite *Psoroptes ovis* is an important ectoparasite of both cattle and sheep. In contrast to *S. scabiei*, it is a non-burrowing mite which feeds off the exudation from skin surfaces (Downing, 1936). Infestation with *P. ovis* results in severe allergic dermatitis in cattle (Stromberg and Fisher, 1986) and scabietic lesions in sheep (Jayawardena et al., 1998). Although vaccination using mite extracts have shown to elicit partial protection in cattle and sheep (Pruett et al., 1998; Smith et al., 2002; Smith and Pettit, 2004), there has been limited success in vaccine development against this species of mite. A subunit cocktail vaccine consisting of seven targets has been tested in sheep with a reduction of more than 55% in mite numbers and lesion size obtained (Burgess et al., 2016).

*Dermanyssus gallinae* (the poultry red mite) is a species that severely affects the poultry egg-laying industry (Van Emous, 2005). Initial vaccination studies with a crude protein fraction of homogenized mites resulted in significant increase in mite mortality compared to control hens (Wright et al., 2009). *In vitro* feeding assays using serum derived from the latter, led to the identification of a serpin (Deg-SRP-1), vitellogenin (Deg-VIT-1), hemelipoglycoprotein (Deg-HGP-1) and a protein of unknown function (Deg-PUF-1) that significantly increased mite mortality rates (Bartley et al., 2015). Field trials comparing soluble mite extract (SME) vaccine to a recombinant vaccine containing Deg-SRP-1, Deg-VIT-1 and Deg-PUG-1 in 384 hens challenged with *D. gallinae* indicated the former vaccine to offer 78% reduction of mites while the latter did not give any significant results (Bartley et al., 2017). This highlights the need for field trials to firmly establish an antigen as being protective. Additional subunit vaccines have been tested, containing tropomyosin, paramyosin (Wright et al., 2016) or subolesin (Harrington et al., 2009). All offered limited protection against infestation.

Taken together the data does indicate a realistic potential for mite vaccines to protect human and animal hosts. Failures or insufficient protection reported to date for mite vaccine studies have been attributed to problems related to formulation, dose, vaccination regime and vaccine delivery (Arlian and Morgan, 2017). Moreover, the parasite-host interaction is rather complex and these and other parasites have documented abilities to evade the host's immune responses through bioactive salivary molecules (Titus et al., 2006; Arlian and Morgan, 2017). Investigating the immunological response to *D. gallinae* infestation revealed that during feeding there is an inhibition of Th1 inflammatory responses (Harrington et al., 2009), a situation echoed by *S. scabiei* infestations (Lalli et al., 2004). It may therefore be beneficial to design future vaccines to elicit a balanced Th1/Th2 response, which may protect the host against infestation.

#### Ticks

Ticks are obligate hematophagous ectoparasites that feed on all classes of terrestrial vertebrates (Sonenshine, 1991). Moreover, ticks are considered very important vectors of diseases affecting cattle and pets, as well as the second most important vector of human diseases after mosquitoes (De La Fuente et al., 2008). Moreover, as hematophagous ectoparasites, the damage inflicted by ticks, especially in livestock, includes the damage to hides, anemia, weight loss and secondary infections (de La Fuente and Contreras, 2015).

The concept of experimental vaccination against ticks has been explored since 1939 when William Trager demonstrated that injecting guinea pigs with whole extracts of *Dermacentor variabilis* tick larvae conferred some protection against subsequent infestations (Trager, 1939). The feasibility of vaccinating cattle against ticks was later demonstrated by Allen and Humphreys (1979). This seminal research led to the discovery of Bm86 in 1986, a protective antigen in membrane fractions of *Rhipicephalus microplus*, identified through classical protein fractionation experiments (Willadsen and Kemp, 1988; Willadsen et al., 1989). This antigen is a membrane-bound glycoprotein located on the gut lumen of the tick digestive tract (Gough and Kemp, 1993) and suggested to be involved in cell-cell or pathogen-gut cell interactions (Liao et al., 2007). The Bm86 (and/or its related homolog Bm95) antigen is the basis for the only commercial tick vaccines developed in 90's, including GAVAC® and GAVAC® Plus available in Latin American countries (Herber-Biotec S.A., CIGB, Camagüey, Cuba), as well as the discontinued TickGARD® and TickGARD®^*PLUS*^ from Australia (Intervet Australia Pty. Ltd., Australia) (De La Fuente et al., 2007), and is still being used effectively on a large scale for vaccination such as performed recently in Venezuela (Suarez et al., 2016). Only one other commercial vaccine is currently produced for the Latin American market by Limor de Colombia® (Bogotá, Colombia) under the product name Go-Tick® or Tick-Vac® that is directed against *R. microplus* infestations (Supplementary Table 1). A probable native vaccine, the manufacturer claims ~80% protection in field tests, though no research publications based on its use have been disclosed to assess the veracity of protection claims.

Vaccination with Bm86-based vaccines results in a reduction of the number of engorging female ticks, their engorgement weight, but mainly their reproductive capacity (Rodríguez et al., 1994). This eventually leads to reduced larval infestation in subsequent generations. However, due to the vaccine's inefficacy against all tick life stages, its variability of protection against different tick strains and species, across geographical regions, and the requirement of several boosts per year for optimal efficacy, the pressing need for improved tick control is reiterated (García-García et al., 1999). The preliminary research that led to the identification of Bm86 illustrated the advantage of vaccinating with protein extracts, which gave a higher vaccine efficacy than the fractionated proteins, probably due to the synergistic effects of protein combinations, proving the feasibility of combinatorial vaccines (Rand et al., 1989; Willadsen et al., 1989). Several studies using Bm86 (Bm86 homologs) or parts thereof in combination with other tick antigens have been published and show promise (Richards et al., 2015; De La Fuente et al., 2016a; Schetters et al., 2016).

A rational approach toward the identification of protective antigens would be to target proteins crucial for the biological function and survival of the parasite including tick attachment to the host, circumvention of the host's defense mechanisms, feeding and digestion of the blood meal, metabolism, mating, fertility, embryogenesis and oviposition (De La Fuente et al., 2016a). To date, several recombinant vaccine candidates identified from different Rhipicephalus species have been validated *in vivo* with their effectiveness in controlling tick populations ranging from 0 to almost 100% (Richards et al., 2015). Some of the most promising candidates currently under investigation include a mix of six peptides (that were identified using reverse vaccinology followed by *in vitro* feeding of peptide-specific antibodies) and aquaporins (Schetters et al., 2016). The latter have been patented by Dr. F. Guerrero and colleagues (US Patent nr(s).: 2013/0315947; US 2016/0361396) for the purpose of vaccine development against ticks as single (Guerrero et al., 2016), as well as combinatorial antigen formulation with a novel gut antigen (Guerrero and De Leon, 2017). Aquaporins have also been found to be effective against *I. ricinus* larvae with the best aquaporin formulation reaching 80% efficacy (Contreras and de La Fuente, 2017).

Other recently tested promising antigens include subolesin vaccine formulations including subolesin/akirin chimera which resulted in a 99% and a 46.4% vaccine efficacy in rabbits against *I. ricinus* and *D. reticulatus*, respectively (Contreras and de La Fuente, 2016). Additionally, recombinant subolesin tested against *H. anatolicum* and *R. microplus* infestation resulted in 65.4 and 54% protective efficacy, respectively (Kumar et al., 2017). The promise of this antigen is evident by the presence of a patent based on a combinatorial vaccine including Bm86 and subolesin (Patent nr.: PCT/EP2014/056248). An improvement of the current vaccine antigen Bm86 via multi-antigen formulations seems a promising avenue to improve an existing effective vaccine.

Although numerous vaccine candidates have been identified, a vaccine capable of protecting against a range of species remains hypothetical. However, in recent studies conducted by Rodríguez-Mallon and colleagues investigated the acidic ribosomal protein P0 (highly conserved among tick species) against *R. microplus* (Rodríguez-Mallon et al., 2015) and *R. sanguineus* (Rodríguez-Mallon et al., 2012). Immunization of rabbits offered an overall efficacy of 90%, mainly via reducing the number of adults and egg hatching (Rodríguez-Mallon et al., 2012). During bovine immunization studies against *R. microplus* and an overall vaccine efficacy of 96% was obtained (Rodríguez-Mallon et al., 2015). As a consequence of these results a vaccine composition based on the P0 peptide was patented recently due to its potential as protective vaccine antigen against a range of ectoparasites (Mallon et al., 2015).

Since the generation of tick sequence databases (genome and transcriptome), *in silico* vaccinology approaches have been used with success to identify protective antigens, as evident from a next generation multi-peptide tick vaccine that has been developed (Schetters et al., 2016). Future studies are now needed to identify protective antigen epitopes to reduce costs associated with production of a commercially viable vaccine. Several other high-throughput techniques including expression library immunization or ELI, sequence suppression subtractive hybridization or SSH, microarray hybridization, RNAi and proteomics have also been evaluated as screening platforms for candidate tick protective antigens (de La Fuente and Contreras, 2015; De La Fuente et al., 2016a; Lew-Tabor and Valle, 2016), with only subolesin being identified. A major limitation in tick vaccine development for livestock is access to pre-vaccination screening tools to identify promising antigens for large-scale production and evaluation in large animal models. In this regard, artificial feeding methods have been developed (Abel et al., 2016; Tajeri et al., 2016; Krull et al., 2017; Böhme et al., 2018), offering some promise as a pre-screening approach with ticks feeding on serum derived from vaccinated hosts (Lew-Tabor et al., 2014; Lew-Tabor and Valle, 2016).

## Metazoan vaccine development: identification to formulation

Significant efforts have been directed toward vaccine development for many metazoan parasites of medical and veterinary importance, since it is generally considered an ideal approach to prevent re-infection/infestation that is generally not achievable with repeated prophylactic chemotherapy (Noon and Aroian, 2017). In successful cases, target product profiles (TPP) or preferred product characteristics (PPC) were developed to establish the value of including a vaccine into public control programs, such as developed for hookworm (Hotez et al., 2013; Bartsch et al., 2016) and schistosomiasis (Molehin et al., 2016) vaccines. These product profiles were refined, not only through successes but also from failures during animal and human trial evaluations. A prime example, comes from hookworm vaccines developed against *A. caninum*, where a native veterinary vaccine based on attenuated L3 larvae failed commercially (Miller, 1978; Schneider et al., 2011) and an abandoned recombinant larval surface protein (ASP-2) that caused undesirable adverse reactions in phase I human clinical trials (Schneider et al., 2011; Bottazzi, 2015). The latter was due to elevated IgE levels elicited within pre-exposed and possibly sensitized test subjects staying within a parasite endemic area causing generalized urticaria (Diemert et al., 2012). It is believed that the IgE axis (including receptors and cellular responses) has evolved to counter infection/infestation by metazoan parasites (helminths and arthropods) that cannot be phagocytosed (Fitzsimmons et al., 2014). Moreover, metazoan parasites can be strong inducers of inflammatory responses (e.g., helminths and mites), and these parasites have also been implicated in human host sensitization and development of various allergies (e.g., tick-induced meat allergies) (Cabezas-Cruz and Valdés, 2014; Fitzsimmons et al., 2014; Steinke et al., 2015; Posa et al., 2017). For helminth infections, a strongly skewed host response toward Th2 immunity is observed and these parasites actively moderate the host Th2 (and other) responses via secreted bioactive compounds that eventually leads to a diminished antiparasitic IgE and cellular response (McSorley et al., 2013; Fitzsimmons et al., 2014; Nutman, 2015). A similar modulation of host defenses toward Th2-mediated anti-inflammatory effects is observed for tick-derived immunomodulatory compounds (Kazimírová and Štibrániová, 2013; Silva et al., 2016). It would appear that Th2 and IgE related responses are vital for natural host antiparasitic defenses (Fitzsimmons et al., 2014). However, protection conferred by recombinant subunit vaccines does not conform to natural anti-parasite immunity and in most cases IgG responses that block essential antigen function mediate protection (e.g., helminths and ticks) (Jonsson et al., 2014; De La Fuente et al., 2016a; Contreras and de La Fuente, 2017; Noon and Aroian, 2017). In general, it seems a fine line exists between positive vaccine therapeutic effects and unwanted hyper-immunity in sensitized individuals that presents an ongoing challenge for vaccine development (Fitzsimmons et al., 2014). Therefore, care in the selection, production and formulation of targets for especially human vaccines must be taken and homology (i.e., structure and epitope) screening of such antigens against libraries of known allergens (i.e., plant and animal) might be a good stage-gate for pre-selection.

In the case of veterinary vaccines, clear product profiles are not as evident. Effective native (e.g., Bovilis Huskvac®, Barbervax®, and GoTick®) and recombinant (e.g., Providean Hidatil EG95®, Cysvax®, Providean Aquatec Sea Lice®, GAVAC®) vaccine preparations have been commercialized, whereas pure recombinant antigens are preferred for human vaccines (Supplementary Table 1). Native vaccine formulations require production of parasite products, often using animal hosts, on a large enough scale to produce a commercially viable product. Therefore, considering the biological complexity of most metazoan parasites, the use of native vaccines (whole organism or purified protein fractions) are generally unfavorable (relative to recombinant vaccines) for commercial vaccine design due to limitations including: scalability of antigen production, high production costs, low vaccine stability and shelf life as well as safety. In some cases, however, native vaccines such as Barbervax® are so effective that they remain more economical, relative to no intervention or mass drug administration MDA (Noon and Aroian, 2017).

### Distilling antigens of interest

**Figure 1:**
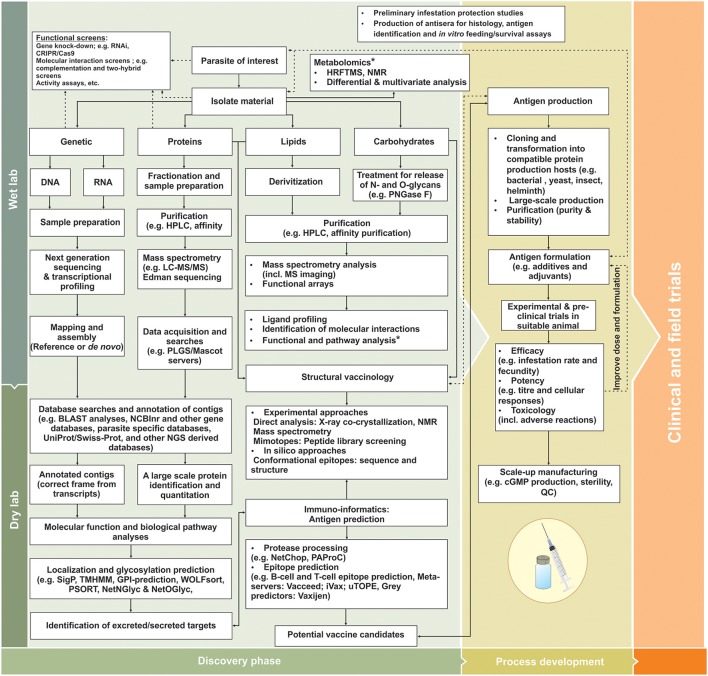
Diagrammatic workflow for identification and evaluation of next generation metazoan vaccine candidates adapted from Haçariz and Sayers (2016). In wet-lab conditions, the parasite of interest is treated to ensure isolation of appropriate factors involved in parasite biology and parasite-host interactions, providing data on genomics, transcriptomics, proteomics, lipidomics, glycomics, and metabolomics levels. During “dry lab” applications, the various parasite components can be analyzed and functionally annotated using various functional and reverse genetics techniques. By employing large-scale techniques and bioinformatics tools, exposed targets able to elicit a host immune response can be preferentially selected and their protective epitopes predicted for improved vaccine design. These targets can enter process developmental stages where antigens are produced and tested in small-scale experimental vaccination trials. Subsequent improvements of protective antigens include vaccine formulation, stability and efficacy during process development, prior to extensive clinical and field trail evaluations. Dashed lines indicate some additional loops for antigen discovery, functional annotation and vaccine improvement and asterisks feedback information from metabolomics studies. cGMP, Current good manufacturing practices; GO, gene ontology; HRFTMS, Fourier transform mass spectrometry; KEGG, Kyoto Encyclopedia of Genes and Genomes; LC-MS/MS, Liquid chromatography-tandem mass spectrometry; MS, mass spectrometry; NMR, Nuclear magnetic resonance; PLGS, ProteinLynx Global; PNGase F, Peptide-N-Glycosidase F; QC, Quality control.

Prior to commercial considerations, identification of antigenic targets remains the essential rate-limiting step for vaccine development. Moreover, proteins that have essential functions in parasite biological processes can be targeted for development of next generation controls and diagnostics that include: host invasion and evasion of immunological responses (also so-called virulence-related proteins); parasite metabolism, development and fecundity; parasite-host co-evolution; even targets related to parasite acquired resistance (i.e., chemotherapeutic resistance) (de La Fuente and Contreras, 2015; Lv et al., 2015; Haçariz and Sayers, 2016; Kuleš et al., 2016; Arlian and Morgan, 2017). However, in many cases parasite proteins identified to date relate mostly to physiological pathways or are stage specific in the parasite and may not necessarily be directly associated with host-parasite interaction. To fill knowledge gaps, complementary technologies that can assist in expanding our current understanding of parasite-host biology and expedite identification strategies to prevent and control parasitic infections/infestations are available (Figure 1). These so-called -omics or systems biology approaches have found fertile ground especially in unicellular parasite/pathogen vaccine discovery and have matured into new emergent fields such as systems vaccinology or immunomics (Hagan et al., 2015; Nakaya and Pulendran, 2015; De La Fuente et al., 2016a; Haçariz and Sayers, 2016; Kuleš et al., 2016; Villar et al., 2017).

In this context, *in silico* comparative approaches are useful for robust identification of additional targets for vaccine development. In the wake of the genomics revolution, sets of sequence data (i.e., genomes, shot-gun, expressed sequence tags and suppression subtractive hybridization or SSH libraries, microarray and RNA sequencing transcriptomic datasets, etc.), representing different life stages and different conditions, have been expanding for many metazoan parasite species of economic importance (Anstead et al., 2015; Greene et al., 2015; Lv et al., 2015; Schwarz et al., 2015; Tyagi et al., 2015; De La Fuente et al., 2016a,b; Kuleš et al., 2016; Arlian and Morgan, 2017; Barrero et al., 2017). These growing repositories of genetic information enable researchers to gain access to a greater complement of molecules involved in parasite and parasite-host biology, also enabling evolutionary analyses to determine parasite diversity and encoded protein conservation/divergence within and between parasitic and non-parasitic species (Lv et al., 2015; Haçariz and Sayers, 2016; Barrero et al., 2017; Mans et al., 2017). Using bioinformatics tools, parasite target sequences can be subjected to a series of analyses such as motif searches (e.g., protein families, domains, conserved catalytic or interaction sites) (Jones et al., 2014), biological pathways and protein interaction network analyses (Kandpal et al., 2009; Khatri et al., 2012; Hernández Sánchez et al., 2016; Nguyen et al., 2016; Rahmati et al., 2017), produce gene ontology (GO) information (Ashburner et al., 2000; Gene Ontology, 2015); predict subcellular localization (Horton et al., 2007) and even predict potential antigens or antigenic regions via reverse vaccinology and immuno-informatics approaches (Maritz-Olivier et al., 2012; Bremel and Homan, 2013; Goodswen et al., 2013, 2017; Maritz-Olivier and Richards, 2014; Lew-Tabor and Valle, 2016) (Figure 1).

Combining such *in silico* methods with manual inspection, literature searches and even additional bioassays, will assist functional annotation of transcripts that lack sequence homology to model organisms (De La Fuente et al., 2016a; Haçariz and Sayers, 2016; Lew-Tabor and Valle, 2016). In this regard, additional reverse genetics approaches such as gene knock-down (e.g., RNAi) and gene editing (CRISPR/Cas9) have been successful in describing *in vivo* protein function for parasitic and non-parasitic (e.g., *Caenorhabditis elegans* and *Drosophila melanogaster*) helminths and arthropods, as well as evaluation of potential targets for parasite/vector control (e.g., expressed library immunization or ELI and phage display) (Dalvin et al., 2009; Ellis et al., 2012; Waaijers et al., 2013; Sandeman et al., 2014; Tröße et al., 2014; Aghebati-Maleki et al., 2016; Britton et al., 2016; De La Fuente et al., 2016a; Zamanian and Andersen, 2016; Crauciuc et al., 2017; Gao et al., 2017; Macias et al., 2017; Rahumatullah et al., 2017).

In the tick research field, systems biology approaches are also gaining momentum with new studies combining -omics approaches (e.g., transcriptomics, proteomics and even metabolomics) to not only define parasite biology and vector-pathogen interfaces, but also identify next generation targets for antiparasitics (Chmelar et al., 2016; Ramírez Rodríguez et al., 2016; Contreras and de La Fuente, 2017). Functional proteomics approaches are currently enjoying a resurgence in parasite research and advances in high-throughput mass spectrometry technologies (including complementary analytical and *in silico* or bioinformatics methods) have improved identification and quantitation of proteins (and protein mixtures), as well as enabled structural analysis of multiprotein complexes (i.e., subunit composition, stoichiometry and topology) (Aebersold and Mann, 2016; Haçariz and Sayers, 2016; Villar et al., 2017). Additional protein and peptide microarrays can provide an *in vitro* platform for functional high-throughput screening of protein targets for protein-protein (i.e., interactomic) and protein-antibody (serodiagnostics and vaccine reactive antigenic epitope) screens (Manzano-Romá et al., 2012; Gaze et al., 2014; Carmona et al., 2015; Driguez et al., 2015; Kassegne et al., 2016). However, this technology remains underutilized in metazoan parasite vaccine research.

A further consequence of proteomic and bioinformatic developments is the emerging field of structural vaccinology (Kulp and Schief, 2013; Donnarumma et al., 2016; Villar et al., 2017; Wang and Chance, 2017). Bespoke antigens can be made that are modeled onto a stabilizing protein scaffold that contains only functional structural epitopes (determined by *in silico* methods and *in vivo* and/or *in vitro* assays) defined from antigen-antibody contacts (via mass spectrometry and crystallographic technologies) that are highly optimized and tailored for specific immune responses (Kulp and Schief, 2013; Malito et al., 2015; Saeed et al., 2017; Simkovic et al., 2017). Such antigens can potentially contain any number of protective epitopes for a number of different strains and species, but this approach has only been investigated for unicellular parasites and pathogens (e.g., Lyme disease vaccine based on the outer surface protein A or OspA) (Kulp and Schief, 2013; Malito et al., 2015). Another major draw-back is the lack of crystal structures (or sufficient homologs) that are available (<1,000, https://www.rcsb.org/) to enable such an approach to vaccine design. In this case *ab initio* molecular modeling could contribute in the absence of defined crystal structures (Khor et al., 2014, 2015, 2017).

A final consideration in metazoan antigen discovery, is the presence of parasites that do not have effective laboratory animal models and/ or require large animal hosts for propagation and challenge, or do not produce comparative responses (including pathological endpoints) between intermediate or definitive hosts (e.g., hookworms) (Schneider et al., 2011; de La Fuente and Contreras, 2015). In these cases, to further antigen discovery, a related parasitic species can be used that have a permissible animal model for vaccine trial evaluations. In this manner homologous target proteins can be identified that may confer cross-species protection such as subolesin/akarin and P0 antigens (Moreno-Cid et al., 2013; de La Fuente and Contreras, 2015; Mallon et al., 2015; Carpio, 2016; Villar et al., 2017). Additional *in vitro* feeding systems for ectoparasites such as ticks, lice, mites and mosquitoes have become valuable tools for parasite study and development of antiparasitics by mitigating the requirement for host animal challenges and affording a platform for high-throughput studies (i.e., infection studies, gene knock-down and chemical assays) (Kessler et al., 2014; Bartley et al., 2015; Sangaré et al., 2016; Agramonte et al., 2017; Kim et al., 2017; Krull et al., 2017; Trentelman et al., 2017). Unfortunately, due to the more intimate and complex association between endoparasitic metazoan helminths, *in vitro* culturing systems are not yet optimal for parasite development of some species (e.g., *H. contortus* and *S. hamatobium*) and culture conditions may not necessarily reflect *in vivo* conditions (a similar case for some ectoparasites) or culture components may interfere with small molecule studies (e.g., peptides and metabolites) (Shepherd et al., 2015; Britton et al., 2016; Driguez et al., 2016). Moreover, success in *in vitro* assays may not guarantee vaccine protection *in vivo* (e.g., poultry red mite) (Bartley et al., 2015, 2017), consequently field trials using the definitive (or equivalent) host animals are still required for final proof-of-concept.

### Production of antigens

Large-scale production of recombinant proteins that maintain immunological activity comparable (or better) to the native parasite protein is another challenge for recombinant vaccine development. Therefore, correct folding and post-translational modification (i.e., glycosylation) will depend upon the protein production host used (prokaryotic vs. eukaryotic). Recombinant protein production in *Escherichia coli* is a popular approach, however, production of insoluble antigens (e.g., *O. ostertagi* rASP1) and unsuccessful application of such proteins in vaccination trials (e.g., OPA and H-gal-GP) have been observed (Vercauteren et al., 2004; Cachat et al., 2010; Matthews et al., 2016). In some cases production of antigens as fusion proteins with maltose binding protein (MBP) and glutathione-S-transferase (GST) in *E. coli* have resulted in excellent protection during vaccination trials, as exemplified by *T. solium* antigens, TSOL16 and TSOL45-1A (in QuilA adjuvant) (Gauci et al., 2012). However, these results might be due to ancillary factors aside from the chosen recombinant antigen that may contribute to the observed protection conferred by native preparations. Moreover, recombinant antigen quality (such as protein solubility, folding and glycosylation) produced by protein production hosts may influence the host immune response (e.g., isoform, specificity and avidity of antibodies produced) (Matthews et al., 2016). To mitigate such outcomes recombinant protein production can be attempted in parasite-derived cells or a closely related species (e.g., *C. elegans* and bacculovirus-insect cell expression systems) in order to produce a protein mimicking the native molecule (Nisbet and Huntley, 2006; Roberts et al., 2013; Hussein et al., 2015; Van Oers et al., 2015). However, for some antigens like *H. contortus* antigen H11, protein recombinantly produced in *C. elegans* was unsuccessful when applied in experimental vaccination trials (Roberts et al., 2013). Moreover, some protective antigens are multiprotein complexes that simply cannot be easily produced using synthetic production hosts. Barbervax® (WormVax, Australia), as an example, is a native vaccine isolated from adult *H. contortis* of which the two major antigenic fractions, H11 and H-gal-GP (~1,000 kDa), consists of multiple enzymes and protein complexes integral to the parasite gut membrane (Supplementary Table 1) (Salle et al., in review).

Protein glycosylation is also regarded as an important factor when manufacturing vaccines as it is known that polysaccharides can serve as a first signal for B cell activation, however, limited glycomics studies have been conducted in metazoan parasites (de La Fuente et al., 2006; Maritz-Olivier et al., 2012; Hokke and Van Diepen, 2017). For ectoparasites such as ticks, preliminary evidence for the importance of carbohydrate moieties in protective antigens was demonstrated by Lee et al. (1991), where protective responses in vaccinated cattle were abolished following treatment of *R. microplus* midgut extracts with sodium metaperiodate. Additional studies, using native and recombinant Bm86 (produced in prokaryotic and eukaryotic protein production systems), showed that the carbohydrate determinants of Bm86 contributed to the protective responses observed in vaccinated animals (Willadsen and McKenna, 1991; de La Fuente et al., 2006). It has also been indicated that tick-derived carbohydrates (such as alpha fucosylation of tick glycoproteins by fucosyltransferases) are role-players in tick-pathogen interactions, such as colonization and transmission in vector cells (e.g., *Anaplasma phagocytophilum*) (de La Fuente et al., 2006; Pedra et al., 2010; Cabezas-Cruz et al., 2017). Similar studies in schistosomes have indicated an important role of glycan epitopes in host-parasite interactions including modulation and evasion of host innate and adaptive immunity, as well as infection biology during snail-schistosome interactions (e.g., fucosylated structures produced on larval surfaces and released during larval transformation and sporocyst development) (Mickum et al., 2014; Jurberg and Brindley, 2015; Smit et al., 2015; Nascimento Santos et al., 2017). Investigation of carbohydrate-protein interactions have been revolutionized with the development of functional glycomics tools such as glycan arrays that have been applied successfully in the analysis of glycan binding protein associated biology, host-pathogen interactions and immune recognition (by antigen specific antibodies) (Heimburg-Molinaro et al., 2011; Rillahan and Paulson, 2011; Arthur et al., 2014). Preliminary studies using glycan arrays have been conducted to identify the repertoire of anti-glycan antibodies produced during helminth infections in both humans and animals (Muthana and Gildersleeve, 2014; Hokke and Van Diepen, 2017). Though identification of glycan antigens has proven to be useful for vaccine or biomarker development (i.e., diagnosis, prognosis, risk prediction, and monitoring immune responses) (Bhatia et al., 2014; Luyai et al., 2014; Yang et al., 2017), application of this technology is still lacking for many metazoan parasites. Additional, structural glycomics techniques such as tandem mass spectrometry (MS), nuclear magnetic resonance (NMR) and compositional (and linkage) analyses of glycoproteins can be used to analyse protein-specific glycosylation, along with traditional blotting and microscopy techniques for localization and distribution studies (Jurberg and Brindley, 2015; Hokke and Van Diepen, 2017).

A separate platform for vaccine antigen delivery/presentation is the use of antiparasitic DNA vaccines, especially for veterinary medicine. These types of vaccine platforms have mostly been applied to protozoal and helminth parasites of medical and veterinary importance with some promising evidence for use in ticks (Ghosh et al., 2007; Guerrero et al., 2014; Wedrychowicz, 2015; Qian et al., 2016; Tebeje et al., 2016; Noon and Aroian, 2017). Currently, only a single plasmid vector has been approved for human DNA vaccine design (Halstead and Thomas, 2011), and a limited number of DNA vaccines have been commercialized against viral pathogens and cancer treatment (e.g., Oncept®, Merial Inc.) in animals (Wahren and Liu, 2014; Finocchiaro and Glikin, 2017). For metazoan parasite vaccines, however, these types of vaccine strategies appear only to be used for initial antigen screening purposes (e.g., expression library immunization) or as pre-clinical evaluations (as either sole or prime-boost strategies) in spite of the availability of newer technologies (e.g., minimized non-viral vectors) (Hardee et al., 2017). Consequently, these strategies appear to be largely abandoned toward final commercialization in favor of recombinant protein vaccines with proper formulations (Ghosh et al., 2007; Merino et al., 2013; Wedrychowicz, 2015; Qian et al., 2016; Tebeje et al., 2016).

### Formulation of vaccines

In general vaccine development, a major shortfall requiring attention is the need for improvement of vaccine immunogenicity and longevity of the immune responses raised (Sinden, 2017). Adjuvants have been employed extensively in vaccine formulations to: reduce the amount of antigen per dose and number of doses required; induce a more rapid immune response; induce broad antibody responses via expansion of B cell diversity; increase the magnitude and functionality of antibodies produced; improve antigen stability; product safety; improve biodegradability, lower costs by improving effectiveness and ease of use (Mohan et al., 2013; Reed et al., 2013; Chauhan et al., 2017b). And with well over 30 defined adjuvant molecules in use today the choice for co-administration of any adjuvant with a chosen antigen is based on a balance between obtaining a higher level of immunogenicity and lesser side effects in the vaccinated host. Some of the adjuvants developed include water-in-oil (e.g., Montanide™), oil-in-water emulsions (e.g., MEtastiM®, Zoetis Inc.), as well as emulsions containing agonists/ligands such as imidazoquinolines (e.g., R848), synthetic oligodeoxynucleotides (i.e., unmethylated CpG motifs), triterpene glycosides or saponins (e.g., Quil-A, ISCOM, QS-21) and monophosphoryl lipid A (MPLA) derivatives (e.g., glucopyranosyl lipid A or GLA) that can tailor host immune responses (i.e., Th1 or Th2) via Toll-like receptor activation (e.g., TLR 7, 9 and 4) (Reed et al., 2013; Chauhan et al., 2017b).

Though many of these additives have been applied successfully in, for example, cattle subunit vaccines (e.g., R848, CpG, and MPLAs) against systemic pathogens and parasites (Rankin et al., 2002; Jones et al., 2013; Reed et al., 2013; Zhou et al., 2014), limited data is available for their use in ectoparasite vaccines. The current commercial helminth vaccines (including formulations used in clinical trials) have been formulated in glucopyranosyl lipid adjuvant either as a stable emulsion (GLA-SE) (i.e., Sm14 and Sm-p80 or SchistoShield®) or as an aqueous formulation (GLA-AF) in combination with alum (i.e., Sm-TSP-2) (Supplementary Table 1). The glucopyranosyl lipid adjuvant is a Toll-like receptor 4 agonist that promotes a strong Th1 (via cytotoxic T lymphocytes) and a balanced IgG1/IgG2 response in vaccinated hosts (Cauwelaert et al., 2016; Dowling and Mansell, 2016). In contrast, for most promising tick-derived vaccine antigens, oil-based emulsions (e.g., Freund's Complete Adjuvant, Montanide™ and saponin adjuvants) have been used for vaccine formulations (García-Garcí et al., 2000; Andreotti et al., 2002; Patarroyo et al., 2002; Canales et al., 2009; Almazán et al., 2010, 2012; Hajdusek et al., 2010; Parizi et al., 2011, 2012; Ali et al., 2015; Schetters et al., 2016). The Bm86-based vaccine GAVAC® formulated in Montanide™ 888 adjuvant, provided superior protection in calf vaccine trials in comparison to yeast produced antigen formulated in saponin and could provide a long-during protection (5–6 months) (Supplementary Table 1) (Rodríguez Valle et al., 2001; De La Fuente et al., 2007). Moreover, a recent study provided additional evidence that formulation of cement cone extracts of *Hyalomma anatolicum anatolicum* in Montanide ISA-50 lowered the dose of antigen required to confer protection in vaccinated goats (Iqbal et al., 2016). The latter adjuvants have been shown to stimulate an enhanced cytotoxic T lymphocyte and antibody response in vaccinated cattle hosts (Dar et al., 2013; Chauhan et al., 2017b).

Unfortunately, adjuvants such as Montanide™ and saponin can cause systemic side effects including tissue damage (Chauhan et al., 2017b), where severe inflammation can “trap” antigens at the injection site preventing a proper host immune response, as well as cause carcass trim losses in production animals such as cattle (Van Donkersgoed et al., 1999; Spickler and Roth, 2003). A pen study using heifers vaccinated with Bm86, also showed adverse reactions to Montanide™ water-in-oil emulsions (Petermann et al., 2017). But since Willadsen and colleagues indicated a significant positive correlation between the size of the injection site reaction and the resulting antibody titer (Willadsen et al., 1995), the authors suggested that a visible local reaction could be linked to better protection in animals to gain acceptance by producers (Petermann et al., 2017).

Some examples of conjugation of antigens to effector molecules to increase antigenicity are also available for metazoan parasite vaccines currently in clinical evaluations, as well as commercial production (Supplementary Table 1) (Hussein et al., 2015; Molehin et al., 2016; Tebeje et al., 2016; Noon and Aroian, 2017). Vaccination studies with sea lice showed that pP0 and pP0Cr chimeric antigens fused to the T-cell epitopes of tetanus toxin, including a fusion protein of measles virus within the same gene construct, delivered an increased protection in comparison to peptides conjugated with KLH alone (Mallon et al., 2015). The latter indicates the need for testing potential protective antigens in a variety of experimental layouts and formulations, using available adjuvant compounds, to optimize their utility for metazoan parasite vaccine development.

Finally, microencapsulation of parasite antigens is a new promising alternative (or complementary) technology to conventional adjuvants that is currently being tested. In this regard, a limited number of studies have explored the use of poly(d,l)-lactide-co-glycolide (PLGA) microspheres as a vehicle for antigen delivery and include native adult worm extracts for *B. malayi* (BmA), *S. haematobium* glutathione S-transferase (P28GST) and a *R. microplus* Bm86-derived synthetic peptide (SBm7462) (Sales-Junior et al., 2005; Saini et al., 2013; Thi et al., 2017). Such microencapsulation of antigens in biodegradable polymers can improve the profile (safety, specificity, and efficacy) of a vaccine candidate by mediating efficient cellular delivery (targeting and uptake), afford different routes of administration (by promoting mucosal adhesion, penetration, and retention), and provide immunostimmulatory or modulatory effects (Himly et al., 2017). The polyesters derived from lactic and glycolic acids (PLGAs) present additional advantages such as targeted release and better dosing, antigen protection and formation of benign degradation products, as well as a reduction in antigen quantity required for protection (Lima and Rodrigues Junior, 1999; Himly et al., 2017). More studies are, however, needed using different encapsulation techniques for both endo- and ectoparasites, including protection parameters (such as vaccine efficacies) to assess whether microencapsulation will play a beneficial role in future commercial metazoan vaccine development. An interesting development in the field is the transformation of a vector-borne pathogen to produce vector-associated antigens to act as a dual live vector vaccine (e.g., *B. bovis* producing a *Haemaphysalis longicornis* glutathione-S-transferase) (Oldiges et al., 2016). Though this approach is still in its infancy, it could provide a new paradigm in vector-borne disease transmission management.

## Challenges and future perspectives

**Figure 2:**
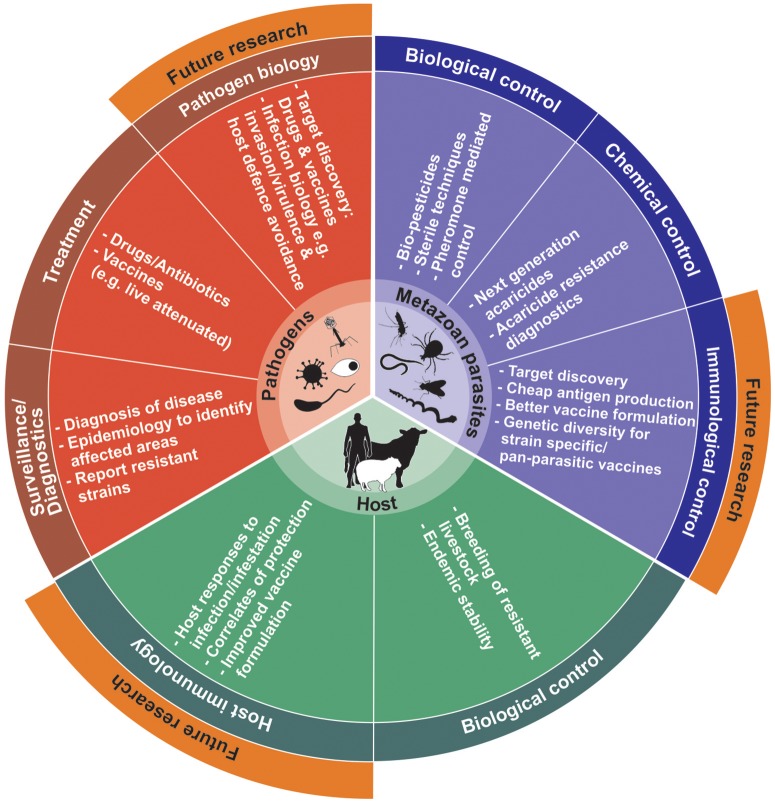
An integrated strategy for parasite and parasite-borne disease control. Several tools are available for management of metazoan parasites (purple), the associated pathogens that can be transmitted (red) and the host animal (green). For metazoan parasite control, pesticides are the most prevalent control measure currently implemented. Resistance to chemical control is a concern that has led to the development of diagnostic tests to identify resistant populations, as well as biological control methods such as bio-pesticides, sterile parasite techniques and pheromones (Yadav et al., 2017). For disease control, treatments are available for most pathogens. Early diagnosis and reports of new and/or resistant pathogenic strains are essential to limit the impact of outbreaks. Some control measures for hosts (human and animal) has involved breeding of resistant stock and/or maintaining endemic stability. Several key areas are currently a priority focus of ongoing research efforts (orange tabs) and these include understanding the host defense against infection/infestation, development of the next generation of vaccines (and diagnostics) and understanding the biology of transmitted pathogens to identify new targets for treatment and diagnosis.

With such a wide array of metazoan parasites of human and veterinary importance, the impact on the health and productivity of the afflicted cannot be underestimated. This problem is compounded with the co-habitation of pathogens with vector parasites that facilitate transmission and spread of debilitating diseases within and between human and animal hosts. Moreover, humans and animals can play host to multiple parasite co-infections (so-called multiparasitism). These interactions can be synergistic or antagonistic, producing diverse effects on host susceptibility, infection duration, transmission profile and clinical manifestations (Thumbi et al., 2014; Vaumourin et al., 2015; Ahmed et al., 2017). Protection through vaccination has become a key research focus area for parasite control as an alternative or complementary approach to costly drug development and growing concerns regarding resistance and chemical residues (Figure 2) (Yadav et al., 2017). However, much information is still lacking for parasitic arthropods such as flies, fleas, lice and mites, but application of new –omics approaches can potentially expand our knowledge on parasite biology and epidemiology, as well as identify new targets for parasite control and serodiagnostic development. For both human and veterinary vaccines, various socio-political (e.g., access, concerns on safety, regulation, and implementation) and scientific (e.g., pathogen and host diversity, host immunosenescence, etc.) challenges remain and will require more attention for the development of future metazoan parasite vaccines (Sheerin et al., 2017). Economics is also a major factor in the deployment of veterinary medicine in particular, as the cost-to-benefit for resource-poor producers may be limited. Therefore, an integrated parasite management strategy is paramount to limit the need and overuse of treatments (i.e., vaccination and chemical prophylactics) that includes: better management practices; selection and breeding of robust or resistant animals, improved biosecurity to limit host exposure, better intervention through diagnostics and therapy, maintenance of herd immunity, etc. (Rashid et al., 2012; Fitzpatrick, 2013; Vreysen et al., 2013; Maqbool et al., 2017; Robbertse et al., 2017; Tabor et al., 2017) (Figure 2).

A combinatorial approach targeting the parasite/pathogen and its arthropod vector via a combination of treatments (including drugs and vaccines), remains a final goal to achieving long-term solutions (Merino et al., 2013; Sinden, 2017). The development of a so-called pan-parasitic vaccine remains hypothetical to date, but if possible can mitigate cost-to-benefit concerns. However, the identification of suitable conserved antigens effective against a multiplicity of metazoan parasitic species is a challenging step and appears to be a product of trial and error. Some proof of principal for such pan-parasitic vaccines have been demonstrated to date for the *Sm*-14, subolesin/akarin and P0 vaccine antigens. As these antigens are all highly conserved intracellular proteins, understanding the mode of action underlying protection via vaccination remains vital. Furthermore, molecular epidemiology and genetic variability studies of parasite populations (i.e., diversity within and between populations) will be needed to assess the conservation of proteins targeted by vaccination and extend vaccine coverage (Sheerin et al., 2017). Moreover, vaccination can influence the life-history traits of pathogens leading to more virulent strains (e.g., *Plasmodium* spp. and viruses) through so-called imperfect vaccines (Gandon et al., 2001; Read et al., 2015). Though evidence for genetic variation (or diversification) has been observed for many metazoan parasites such as helminths (especially on pesticide resistance), the long-term effects of vaccination on the evolution of metazoan parasite life-history traits are not clear and require further investigation (Kennedy and Harnett, 2013).

As we move into the cosmos of an integrated veterinary and human health paradigm (Yamey and Morel, 2016; Xie et al., 2017), a renewed and concerted effort is needed to create metazoan vaccines that are relatively simple to produce, correctly formulated and structurally stable, containing conserved epitopes for cross-species vaccination, with the right qualities for commercialization and public distribution. Therefore, establishment of cooperatives and consortia (Supplementary Table 2) that combines the skills of a myriad of disciplines (such as economics, mathematics, social sciences, veterinary and medical sciences, chemical and industrial engineering, etc.), as well as buy-in from industry and governmental institutions (including national and international veterinary and human health organizations), is required to address in full the needs of vaccine development in the next millennium.

## Author contributions

CS conceived and coordinated the writing of this publication. CS, SR, MF, SB contributed to literature research and writing. CS, SR, CM-O contributed in critical review and revision of the entire manuscript prior to submission.

### Conflict of interest statement

The authors declare that the research was conducted in the absence of any commercial or financial relationships that could be construed as a potential conflict of interest.

## Footnotes

^1^Organization, W. H. Available: http://www.who.int/neglected_diseases/diseases/en/ [Accessed 2017].
